# The value of visual field testing in the era of advanced imaging: clinical and psychophysical perspectives

**DOI:** 10.1111/cxo.12551

**Published:** 2017-06-22

**Authors:** Jack Phu, Sieu K Khuu, Michael Yapp, Nagi Assaad, Michael P Hennessy, Michael Kalloniatis

**Affiliations:** ^1^ Centre for Eye Health The University of New South Wales Kensington New South Wales Australia; ^2^ School of Optometry and Vision Science The University of New South Wales Kensington New South Wales Australia; ^3^ Department of Ophthalmology Prince of Wales Hospital Randwick New South Wales Australia

**Keywords:** Bloch's law, glaucoma, optical coherence tomography, perimetry, psychophysics, Ricco's law, spatial summation, structure‐function, temporal summation, tilted disc syndrome

## Abstract

White‐on‐white standard automated perimetry (SAP) is widely used in clinical and research settings for assessment of contrast sensitivity using incremental light stimuli across the visual field. It is one of the main functional measures of the effect of disease upon the visual system. SAP has evolved over the last 40 years to become an indispensable tool for comprehensive assessment of visual function. In modern clinical practice, a range of objective measurements of ocular structure, such as optical coherence tomography, have also become invaluable additions to the arsenal of the ophthalmic examination. Although structure‐function correlation is a highly desirable determinant of an unambiguous clinical picture for a patient, in practice, clinicians are often faced with discordance of structural and functional results, which presents them with a challenge. The construction principles behind the development of SAP are used to discuss the interpretation of visual fields, as well as the problem of structure‐function discordance. Through illustrative clinical examples, we provide useful insights to assist clinicians in combining a range of clinical results obtained from SAP and from advanced imaging techniques into a coherent picture that can help direct clinical management.

The visual field broadly refers to the area in which a stimulus can be visually detected.^1^, ^2^ From the point of fixation, the monocular visual field of a normal human observer extends approximately 50 degrees superiorly, 70 degrees inferiorly, 60 degrees nasally and 100 degrees temporally.^2^, ^3^, ^4^ The visual field can be measured using a variety of perimetric techniques.^5^, ^6^ The extent and shape of the visual field varies with stimulus parameters, such as stimulus size.^5^, ^6^ In normal observers, kinetic perimetric thresholds coincide with the underlying spatial location of a static threshold obtained using the same stimulus size and luminance.^7^

In clinical practice, standard automated perimetry (SAP) is a common method of assessing the visual field,^8^, ^9^, ^10^ becoming increasingly popular in clinical practice and research settings since the 1970s and 1980s.^11^ As visual field results can provide clues regarding the location of the anomaly along the visual pathway, it is an instrumental component of the ocular and neurological examination;^12^, ^13^, ^14^, ^15^, ^16^, ^17^ however, recent studies have highlighted a number of problems with visual field testing in clinical practice. For example, the frequency of performing visual field assessment for diseases such as glaucoma is often not carried out uniformly across eye‐care practitioners.^18^, ^19^ There may also be structure‐function discordance within the examination results, whereby the defects found on structural measurements do not correlate well with SAP,^20^, ^21^ such as in pre‐perimetric^22^, ^23^ (also known as ‘mild’^24^) glaucoma. A number of models have been suggested to explain this discordance (reviewed in Malik, Swanson and Garway‐Heath^25^). Concurrently, there is increasing interest in objective, quicker and repeatable imaging techniques such as optical coherence tomography (OCT),^26^ which may help to obtain clinical data that do not rely on the subjective responses of the patient, particularly in the earlier stages of disease.^27^

This review paper contains a number of clinical examples of patients seen at the Centre for Eye Health^28^, ^29^, ^30^ to illustrate the role of visual field testing using SAP in a modern era of advanced imaging techniques. All patients had given their written informed consent for use of their anonymised results, with ethics approval given by the relevant University of New South Wales Human Research Ethics Committee. Research visual field results in the relevant figures were conducted in accordance with the tenets of the Declaration of Helsinki. Cases displaying structure‐function concordance and discordance are illustrated. Structure‐function discordance is put into the context of a number of construction, design and psychophysical principles behind SAP. A number of recent studies that have challenged current visual field testing paradigms are discussed, which may be promising in reconciling structure‐function discordance.

## STANDARD AUTOMATED PERIMETRY IN CLINICAL PRACTICE

SAP is a non‐specific term used to describe any perimetric test measuring the detection threshold of a static, achromatic light stimulus of fixed size (Goldmann size III, GIII), presented for a fixed duration (approximately 100 to 200 ms) upon an achromatic background of constant luminance (1–10 cd/m^2^). Output measurements of SAP are typically provided using decibel (dB) scaled units, which are not measures of luminance intensity but rather of the attenuation of light from the instrument's density filters. A ‘high’ dB value means that the patient has responded to a highly attenuated – or dim – light stimulus. Output dB values are specific to the individual instrument, based on its maximum output stimulus and background luminance and hence, dB values are not directly comparable across SAP instruments.^9^, ^31^ Recent studies have provided conversion factors for dB into luminance values, based on instrument‐specific maximal and background luminances.^5^, ^32^

As SAP is widely used in clinical practice and research settings, it is often the reference for which other forms of perimetry are compared.^15^ Several different instruments have been devised which purportedly target different visual functions that may be affected in early disease.^33^, ^34^, ^35^, ^36^ There is debate as to whether certain types of retinal ganglion cell or visual pathways may^37^, ^38^, ^39^ or may not be^40^, ^41^, ^42^, ^43^, ^44^ differentially affected in early stages of disease. For example, new objective‐based techniques such as OCT,^45^, ^46^, ^47^ different forms of visual function assessment^48^, ^49^ and alternative SAP algorithms^50^, ^51^ have shown some promise in detecting deficits in visual functions other than contrast sensitivity thresholds, particularly in pre‐perimetric glaucoma. Although studies suggest that SAP may be relatively insensitive to early visual field changes in patients with disease, there exist no other widely accepted alternatives in clinical practice (see Jampel and colleagues^15^ for a full review). One of the key limitations of selective perimetry is the lack of evidence that specific and unique visual functions and pathways are tested.^52^

In clinical practice, the reliability of SAP results is affected by a range of factors, including those that are patient‐related. Modern thresholding algorithms have been developed to increase test efficiency and reduce patient fatigue (see McKendrick^53^ for a full review and select recent papers for newer algorithms^54^, ^55^, ^56^). The reliability of the visual field results can be assessed using four primary indices or some variation thereof: fixation losses, false positives, false negatives and the results of gaze tracking. The manufacturer of the SAP instrument often provides a cut‐off value for flagging a result as unreliable,^8^, ^57^ although such values in the literature are variable, ranging anywhere between 15 and 33 per cent.^22^, ^58^, ^59^, ^60^, ^61^, ^62^, ^63^ Inconsistencies have been suggested to be due to a range of factors, such as different patient populations, cultures, languages, educational backgrounds and understanding, visual field loss and technician ability, although these cut‐offs are also thought to be arbitrary.^64^

**Figure 1:**
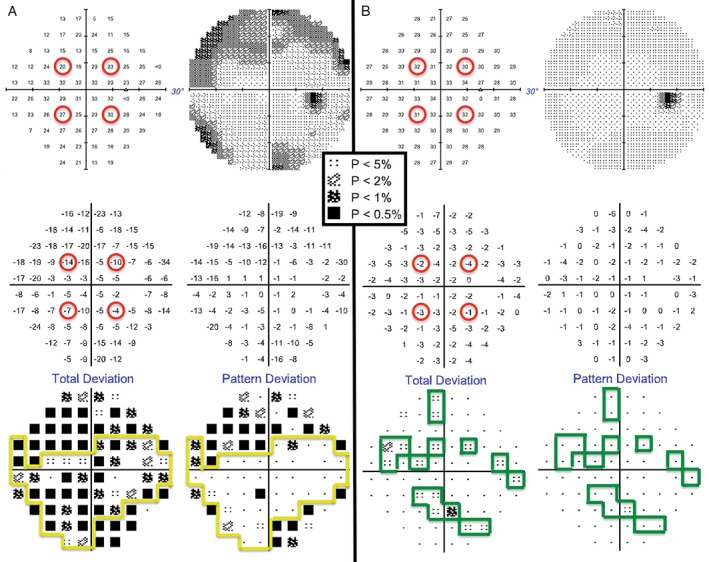
The right eye Humphrey Field Analyzer (HFA) 30–2 SITA‐Standard visual field results for a 13‐year‐old Asian female (top left: thresholds [dB]; top right: greyscale; middle left: difference in dB from normative database; middle right: difference in dB from normative database after subtracting the patient's Hill of Vision [HoV]; bottom left: ‘Total Deviation’ plot, based on the values in the middle left, with probability scale of normality; bottom right: ‘Pattern Deviation’ plot, based on the values in the middle right with probability scale of normality). It was the first time she had undertaken visual field testing (A) and she did not have a good understanding of the task, leading to errors in establishing the initial HoV with the four seeding points (upper left, middle left). After practice and improved task understanding, thresholds at the four seeding locations improved (B). Minor depressions of low significance only appeared in the ‘Total Deviation’ plot (B, lower left) and once the HoV was considered, there was a single significant defect on the ‘Pattern Deviation’ plot (B, lower right). A key for the greyscale levels of probability of normality is shown as an inset.

**Figure 2:**
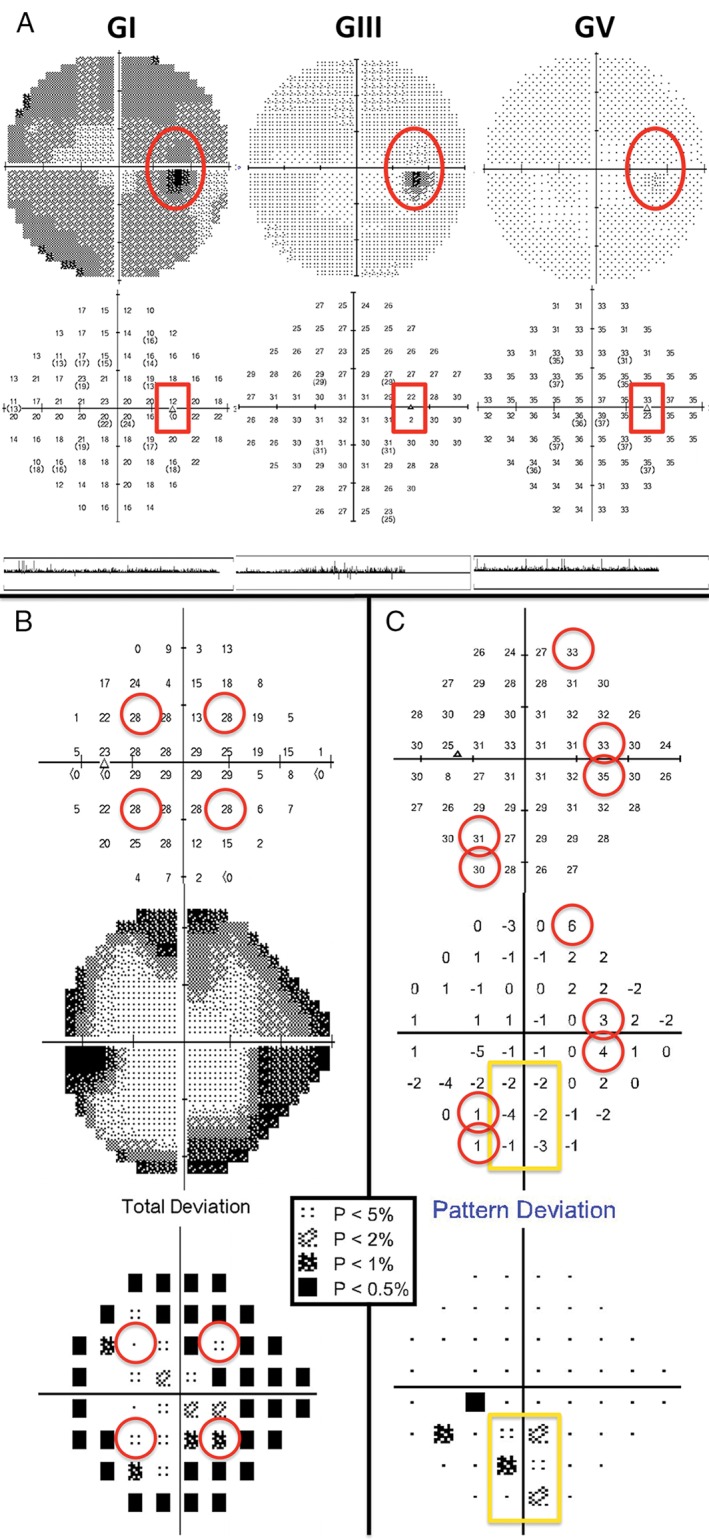
Reliability measurements in visual field assessment. (A) Errors in blind spot mapping can occur depending on the stimulus size and optic disc size and morphology (Humphrey Field Analyzer [HFA] 30–2 full threshold visual field results: top, greyscale; middle, thresholds [dB]; bottom, gaze tracker. Fixation loss percentages were: Goldmann size I [GI], zero per cent, Goldmann size III [GIII], five per cent and Goldmann size V [GV], 100 per cent). The two test locations marked in the red boxes and by the dark spots on the greyscale are excluded from analysis as they correspond to locations where the blind spot may be tested by the instrument. In the case of GI, the lower of the two points was noted to have less than zero dB for a threshold, while there was no absolute scotoma found with GIII or GV at the same location. In particular, testing using a GV stimulus in this patient could not accurately map the blind spot (100 per cent fixation losses, in comparison to zero fixation losses using GI and five per cent fixation losses using GIII) and fixation had to be monitored using the gaze tracker. (B) A cloverleaf‐type (also known as ‘Mickey Mouse ears’) defect in a patient whose attention waned with increasing test duration. The initial four seeding points (red circles) exhibited only mildly depressed sensitivity or were near normal on the raw threshold map (top) and ‘total deviation’ plot (bottom). Surrounding peripheral points showed more significant depressions, especially on the greyscale plot (middle). This patient exhibited false negative errors of 46 per cent. (C) Higher sensitivity is indicative of a trigger‐happy patient. Manual examination of raw threshold values (top) can reveal these points (red circles), which shift the patient's Hill of Vision higher (middle), producing artificial flagged points on the ‘Pattern Deviation’ plot (yellow box, bottom). A key for the greyscale levels of probability of normality is shown as an inset.

Patient attention and task understanding play important roles in test reliability, for example, to maintain fixation and to respond to very dimly seen stimuli, while refraining from responding to the absence of stimuli (Figure 1).^65^ There is a significant practice effect in performing visual fields. Errors from novice patients include inattention to latter parts of the test or trigger‐happy behaviour.^8^ Altered sensitivities in regions may alter the patient's ‘Hill of Vision’ and thus, erroneously flag adjacent points of normal or abnormal sensitivity (Figure 2).

False negatives may also be elevated in patients with visual field defects, due to irregular contrast sensitivity and sampling by the underlying retinal sensory elements and/or circuitry,^66^, ^67^, ^68^, ^69^ particularly when threshold sensitivities fall below certain levels.^70^, ^71^ False negative rates in patients with glaucomatous visual field loss may be as high as 42 per cent in glaucoma compared to less than 20 per cent expected in normal observers.^66^ Larger stimulus sizes (for example, Goldmann size V, GV) and the high luminance level at which the stimulus is presented at the blind spot may encroach upon the adjacent retinal regions and increase stray light, thereby elevating the proportion of apparent fixation losses.^72^, ^73^ Therefore, gaze trackers are useful, when paired with the proportion of fixation losses to determine if it is due to patient‐related factors of poor fixation or trigger‐happy behaviour or due to instrument‐related factors, such as stimulus size or incorrect initial blind spot mapping due to atypical disc physiology (Figure 2).^74^ Inconsistencies in visual field reliability indices are problematic for directly translating visual field results between studies and applying cut‐offs in clinical practice. As visual field testing is used for assessment of ocular and neurological diseases, further studies into the reliability characteristics of patients with different pathological conditions are required to determine optimum cut‐offs for accurate interpretation.

## TEST PARAMETERS USED IN STANDARD AUTOMATED PERIMETRY

### Background luminance and pupil size

The Humphrey Field Analyzer (HFA) and the recent Octopus perimeters (for example, Octopus 600 and 900) use a background luminance of 10 cd/m^2^, which renders the adaptive state of the eye to be within the low photopic range of vision. A relatively lower background luminance (for example, Octopus 101 model, 1.27 cd/m^2^ or the Medmont Perimeter, 3.2 cd/m^2^) can render the adaptive state of the eye to be within the high scotopic or mesopic range, depending upon pupil size. This is problematic because the cone and rod pathways have been shown to respond and interact differently to contrast, resulting in different perceptual experiences.^75^ Although relatively dimmer backgrounds have been suggested for examining patients with ocular disease with impaired dark adaptation,^76^, ^77^, ^78^ a lower background luminance means that some observers are tested within the non‐linear section of the threshold‐versus‐intensity (TVI) curve (the function relating contrast threshold and background luminance).^79^, ^80^, ^81^, ^82^ Testing within Weber's law, where contrast remains constant despite changes to background luminance requires approximately 100 Trolands (Td) of retinal illuminance.^83^, ^84^ This is important to eliminate the effects of background fluctuations in quanta, which may produce inconsistent threshold responses.^79^, ^85^ For example, within the mesopic adaptation range (the de Vries‐Rose section of the TVI curve) thresholds are related to the square root of background luminance and detection is limited by quantal fluctuations.^86^, ^87^ Due to the different sections of the TVI curve, pupil size is also an important consideration. A background luminance of 10 cd/m^2^ requires a pupil size of roughly 3.5 mm in diameter to meet the cut‐off of 100 Td to test within the Weber slope.^83^, ^84^ In comparison, a background luminance of 1.27 cd/m^2^ requires a pupil diameter of 10 mm.

A luminance within the low, rather than high, photopic range can be more comfortable for the patient and reduce artificial pupillary constriction. Decreases in retinal illumination can also be caused by media opacities, such as cataract, which produces a characteristic generalised reduction in sensitivity.^88^ It is recommended to maintain a consistent pupil size that renders adaptation within the Weber slope for visual field testing.

### Stimulus size and duration: summation characteristics

Summation describes the ability of the eye to sum individual quanta of light over time (temporal) or over an area (spatial). The relationship between luminance, area and stimulus duration is expressed mathematically by: *L.A^n1^.t^n2^ = k* (where *L* is the luminance of the stimulus, *A* is the stimulus area, *t* is the stimulus duration, *n1* and *n2* represent the spatial and temporal summation exponents, respectively, and *k* is a constant).^89^ From this equation, Ricco's law of spatial summation (*L.A^n1^ = k*) and Bloch's law of temporal summation (*L.t^n2^ = k*) can be derived.^90^, ^91^ When *n* is equal to one, the test stimulus is operating within the critical area (Ac) or critical duration (Tc) of complete summation and there is a linear relationship between luminance and stimulus duration; outside of Tc or Ac, this relationship is non‐linear.^5^, ^6^, ^92^, ^93^, ^94^, ^95^, ^96^, ^97^

Although both size and duration are important considerations in perimetric testing, these are fixed in commercial standard automated perimetry. A brief stimulus presentation (100 to 200 ms in SAP) is below the minimum latency of a voluntary saccadic eye movement and above Bloch's critical duration of temporal summation (Tc).^98^ The standard GIII (diameter of 0.43 degrees) stimulus maximises the dynamic range of the instrument (by up to 12 dB in the periphery^99^), allows more reliable thresholds to be obtained compared to smaller sizes,^5^, ^100^, ^101^, ^102^, ^103^ and is less susceptible to blur.^6^, ^104^ A summary of Goldmann stimulus sizes available on the HFA is listed in Table 1.^6^, ^105^, ^106^

**Table 1:** Stimulus sizes used in standard automated perimetry. Goldmann size designations are listed with the stimulus diameter in degrees, area and in log area.

Goldmann sizedesignation	Diameter(degrees)	Area(degrees^2^)	Log area (logdegrees^2^)
I	0.11	0.009	−2.036
II	0.22	0.037	−1.433
III	0.43	0.147	−0.831
IV	0.87	0.590	−0.229
V	1.73	2.358	0.373

Several studies have suggested that use of a stimulus outside of Ac or Tc does not yield the maximum threshold elevation in a region affected by disease, in comparison to when a stimulus size is operating within complete summation.^32^, ^94^, ^95^, ^97^, ^107^ Although stimulus parameters used in SAP are said to be historical precedents,^108^ there are limitations in instrumentation^109^ and dynamic range^110^ that render the optimisation of size and duration an area of ongoing research.

## TEST PATTERNS IN STANDARD AUTOMATED PERIMETRY

**Figure 3:**
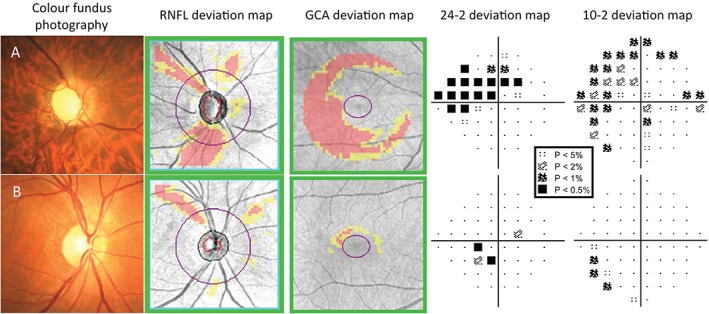
Examples of patients where a 10–2 visual field has helped to determine the extent of the central visual field defect found on the 24–2 (Humphrey Field Analyzer [HFA] SITA‐Standard). A key for the greyscale levels of probability of normality is shown as an inset. (A) The right eye findings of a 54‐year‐old Asian man with moderate normal‐tension glaucoma. The disc size was average, with enlarged vertical cup. There was evidence of inferior neuroretinal rim thinning, with corresponding retinal nerve fibre layer (RNFL) loss on the deviation map and ganglion cell‐inner plexiform layer (GCIPL) thinning on the Ganglion Cell Analysis (GCA) deviation map. The 24–2 visual field showed a superior nasal step defect extending in an arcuate fashion, with points encroaching upon fixation. 10–2 visual field showed the central defect in greater detail, with reductions in sensitivity as near as one degree from fixation. (B) A 46‐year‐old Caucasian man with previous ischaemic attack resulting in superior RNFL loss, as seen in both the fundus photograph and the RNFL deviation map. Although the GCA deviation map showed little significant reduction in GCIPL thickness, the 24–2 visual field deviation map showed points of reduced sensitivity within 10 degrees of fixation. The 10–2 visual field showed that the reduction in sensitivity was located mainly seven to nine degrees from fixation and not within the central five degrees.

Early studies have suggested that the majority of significant visual field defects occur in the central 30 degrees from fixation.^111^, ^112^ Grid patterns examining this region eventually became widely used and standardised, such as the 10–2, 24–2 and 30–2 test grids on the HFA^58^, ^59^, ^113^, ^114^ (denoting the approximate extent of the visual field examined and the amount of spacing from the horizontal and vertical midlines^8^). Recent studies^115^, ^116^, ^117^ have examined the role of different test patterns and densities for a range of diseases; for example, use of the 10–2 pattern to sample the central visual field in glaucoma. For example, the 10–2 pattern has a finer point density compared to 24–2 and 30–2^118^, ^119^, ^120^ and is able to better sample the central papillomacular bundle. Points may be flagged as having reduced sensitivity using a typical 24–2 or 30–2 test grid with six degrees test point spacing, but the true extent of the defect may be missed unless a denser sampling grid, such as a 10–2 with two degrees point spacing, is used (Figure 3). This may be useful in certain types of glaucoma that have been suggested to show more progression in the central visual field,^121^ although whether or not the type of glaucoma is important for the location of defect is debated.^122^ Combinations of test patterns have also been suggested to achieve adequate test density^123^, ^124^ and customisation and sampling of specific regions of interest have been suggested to increase test efficiency.^125^, ^126^, ^127^, ^128^

## THE STRUCTURE‐FUNCTION RELATIONSHIP IN VISUAL FIELD TESTING

Diseases affecting various regions along the visual pathway, from the retina up to the cortex, produce different types of visual field defect. Such defects are described by location (for example, centrocaecal, arcuate, quadrantonopia, hemianopia), by depth (relative or absolute scotoma), completeness (partial or full) and congruity (similarity between the two eyes). The monocular and binocular location of the defect can help determine the affected anatomical location. Generally, increasing congruity indicates a defect that is located in higher cortical areas. Therefore, visual field examination can localise structural deficits and determine the extent of underlying structural damage.

### Ocular media

**Figure 4:**
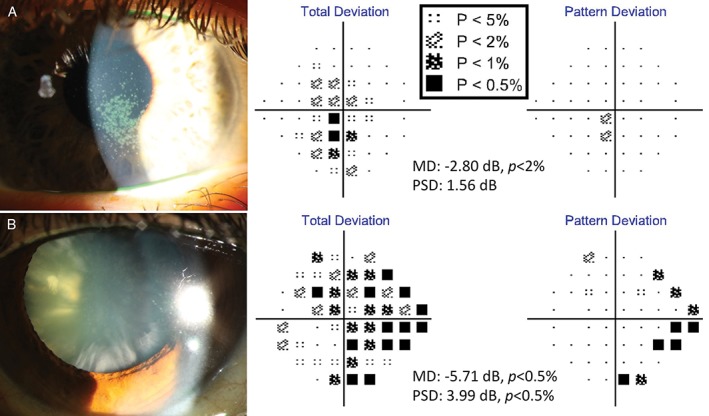
Examples of media opacity visual field defects (Humphrey Field Analyzer [HFA] 24–2 SITA‐Standard). (A) The visual field result of a 38‐year‐old Caucasian woman with severe dry eye manifesting as confluent central corneal superficial punctate epitheliopathy. There were significant central total deviation defects with accompanying mean deviation (MD) value flagged at p < 0.02 (−2.80 dB). Some regions of superficial punctate epitheliopathy were dense enough to result in pattern deviation (PD) map defects; however, these focal defects were not numerous or dense enough to result in a significant pattern standard deviation (PSD) value (1.56 dB, p > 0.10). (B) The visual field result of a 63‐year‐old Asian woman with significant mixed cataracts. Her visual acuities were 6/12^−2^ R and L. In particular, there was a number of dense cortical spoke cataracts. The diffuse defects on the total deviation map were characteristic of a generalised media opacity, while the focal depressions located primarily in the periphery of the PD map were mainly due to the relatively dense peripheral cortical cataracts. As expected, MD scores were depressed at −5.71 dB (p < 0.005) and the extent of PD defects were enough to also flag the PSD score (3.99 dB, p < 0.005). A key for the greyscale levels of probability of normality is shown as an inset.

Global measurements of visual field sensitivity such as the mean deviation (MD) and total deviation (TD) map on the HFA can be affected by medial opacities.^88^ Common examples of these artefacts include dry eye and cataracts (Figure 4). Contact lenses can cause some artefacts due to dry eye or altered optical properties such as from multifocal lens designs.^129^ Such depressions are typically diffuse on the TD map and correlate with the location of the media opacity, although opacities that are sufficiently deep may also result in concurrent defects on the pattern deviation (PD) map.

### Retinal pathology

**Figure 5:**
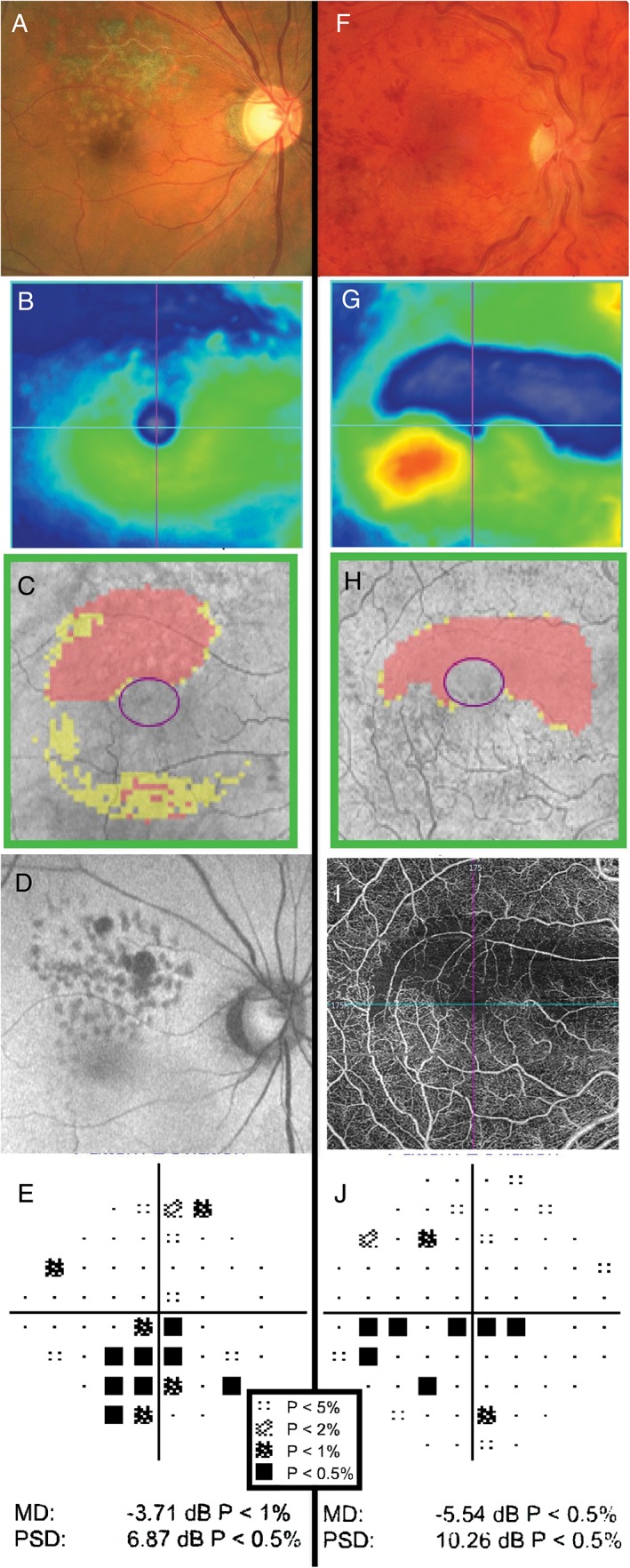
Examples of retinal pathology causing visual field defects. (A–E) The clinical findings of a 68‐year‐old Asian man who had previously undergone retinopexy and intravitreal anti‐vascular endothelial growth factor injections for right branch retinal vein occlusion. The Cirrus optical coherence tomography (OCT) macular thickness heat map (B) and Ganglion Cell Analysis (GCA) deviation map (C) show reductions in neural tissue in the superior macular region. Autofluorescence (D) also highlights the area of atrophy. In (E), the pattern deviation map from the Humphrey Field Analyzer (HFA) 24–2 SITA‐Standard visual field shows structure‐function concordance with focal depressions in the inferior field. (F–J) The clinical findings of a 29‐year‐old Caucasian man with multiple previous retinal vein occlusions in the right eye secondary to Factor V Leiden hypercoagulability. Dilated fundus examination showed diffuse and widespread haemorrhages, dilated and tortuous retinal veins and optic disc oedema in the right eye (F). Cirrus OCT heat map showed oedema of the inferior macula and thinning of the superior macula (G). Similarly, the GCA deviation map showed thinning superiorly (H). OCT‐angiography imaging showed loss of underlying vasculature in the superior region of thinning, indicative of ischaemia (I). This explained the presence of a clear inferior visual field defect (HFA 30–2 SITA‐Standard) adjacent to fixation and thus structure‐function concordance (J). A key for the greyscale levels of probability of normality is shown as an inset.

Overt retinal diseases, such as retinal vascular occlusion or retinal degenerations, are typically accompanied by correlating functional defects in the visual field (Figure 5). The depth of the defect may depend on a range of factors, such as the extent of the underlying structural loss and the duration since onset. Advanced imaging techniques such as OCT can determine the extent of tissue loss and which retinal layers are affected. Separation or loss of certain retinal layers can give hints to whether a visual field defect is expected to be relative or absolute. Modalities such as autofluorescence and OCT angiography can also delineate the expected boundary of the visual field defect but subtle changes in vasculature have yet to yield significant^130^ structure‐function relationships, such as in glaucoma.^131^

When the extent of the visual field defect extends beyond the central retina or if small islands of vision are affected or spared in the periphery such as in retinitis pigmentosa, kinetic perimetry is likely to be better than SAP at measuring visual function.^130^, ^132^ Although some SAP instruments also include the ability to perform kinetic perimetry, such as the HFA‐3, the extent of the measurable visual field is still limited by instrument design (for example, up to 42 degrees superiorly for the HFA‐3).

### Optic nerve pathology

**Figure 6:**
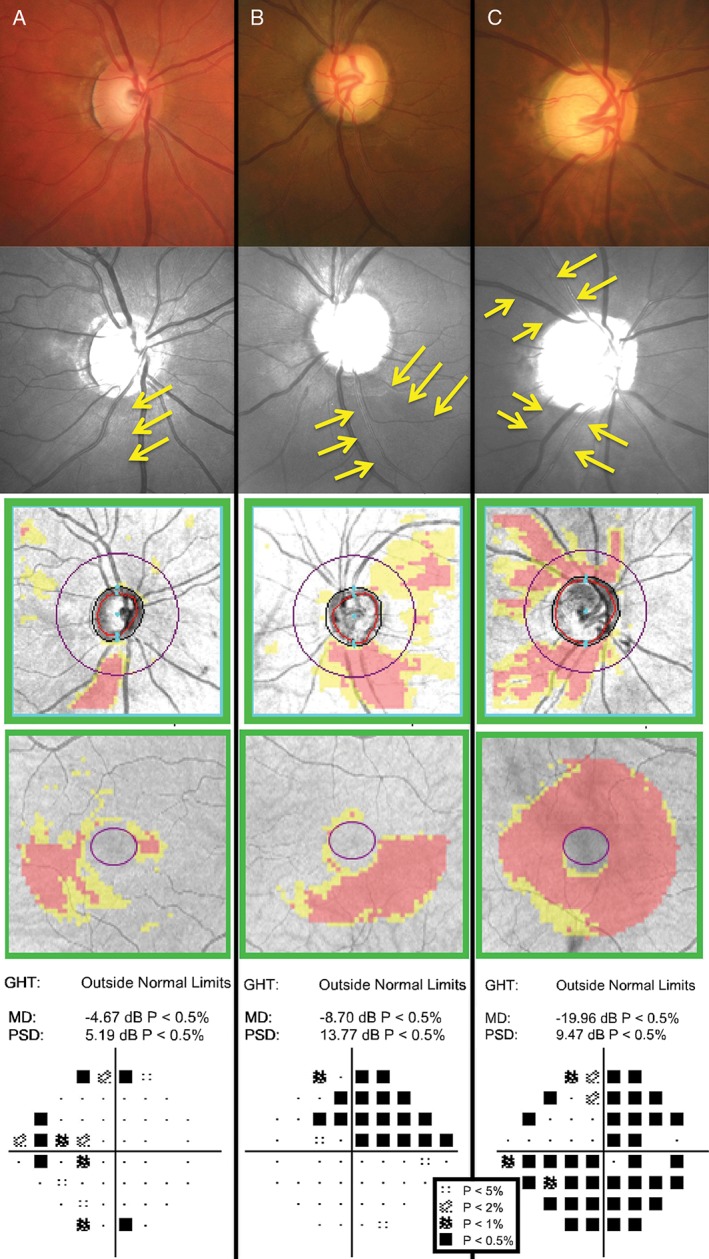
Examples of different stages of glaucoma designated by Mills and colleagues,^133^ with (top to bottom) colour fundus photographs, green‐filtered (red‐free) fundus photographs (yellow arrows indicate areas of retinal nerve fibre layer [RNFL] drop out), Cirrus optical coherence tomography (OCT) RNFL deviation map, Cirrus OCT Ganglion Cell Analysis (GCA) deviation map and Humphrey Field Analyzer (HFA) 24–2 SITA‐Standard pattern deviation map. A key for the greyscale levels of probability of normality is shown as an inset. (A) The right eye findings of a 49‐year‐old Caucasian man with early high‐tension glaucoma. Inferior optic nerve head thinning with corresponding RNFL loss on the deviation map showed structure‐function concordance with the nasal step defect. His HFA mean deviation (MD) score was −4.67 dB (p < 0.005) and pattern standard deviation (PSD) score was 5.19 dB (p < 0.005). (B) The left eye findings of a 60‐year‐old Caucasian woman with moderate high‐tension glaucoma. A predominantly inferior, wide wedge RNFL defect was accompanied by a corresponding superior arcuate defect extending from the nasal region. Her HFA MD score was −8.70 dB (p < 0.005) and PSD score was 13.77 dB (p < 0.005). (C) The right eye findings of a 78‐year‐old Hispanic man with chronic, advanced narrow angle glaucoma. In spite of the large disc, fundoscopic examination showed deeply‐cupped optic nerve head, with almost complete loss of the superior and inferior neuroretinal rim. As expected, there were both superior and inferior arcuate‐type defects across the visual field. Approximately 75 per cent of all points were flagged as below the five per cent level of normality. His MD score was −19.96 dB (p < 0.005). Interestingly, his PSD score was 9.47 dB (p < 0.005), which is apparently ‘better’ than the result in the patient in (B). As PSD is a standard deviation score considering all points across the visual field, the score was not as low as that of the patient in (B) because there are more points of reduced sensitivity.

Glaucoma uniquely offers the opportunity to examine the structure‐function relationship because of the way the retinal nerve fibre layer and optic nerve are affected at discrete locations. National bodies and guidelines recommend the use of SAP for diagnosis and monitoring of patients with glaucoma.^15^, ^16^ SAP results, commonly mean deviation values, are used for staging and monitoring for progression and hence, guidance of management (Figure 6).^58^, ^133^, ^134^ Typically, some variation of the 24–2 test pattern is used in conjunction with an adaptive thresholding algorithm, as it affords a balance of reducing variability (the more peripheral points used in the 30–2 pattern are excluded^135^), test time and fatigue,^136^, ^137^ while testing the nasal and central regions of interest, where glaucomatous defects commonly occur.^138^, ^139^ A variety of structure‐function maps are used in research settings^140^, ^141^ and are commercially available.^142^

**Figure 7:**
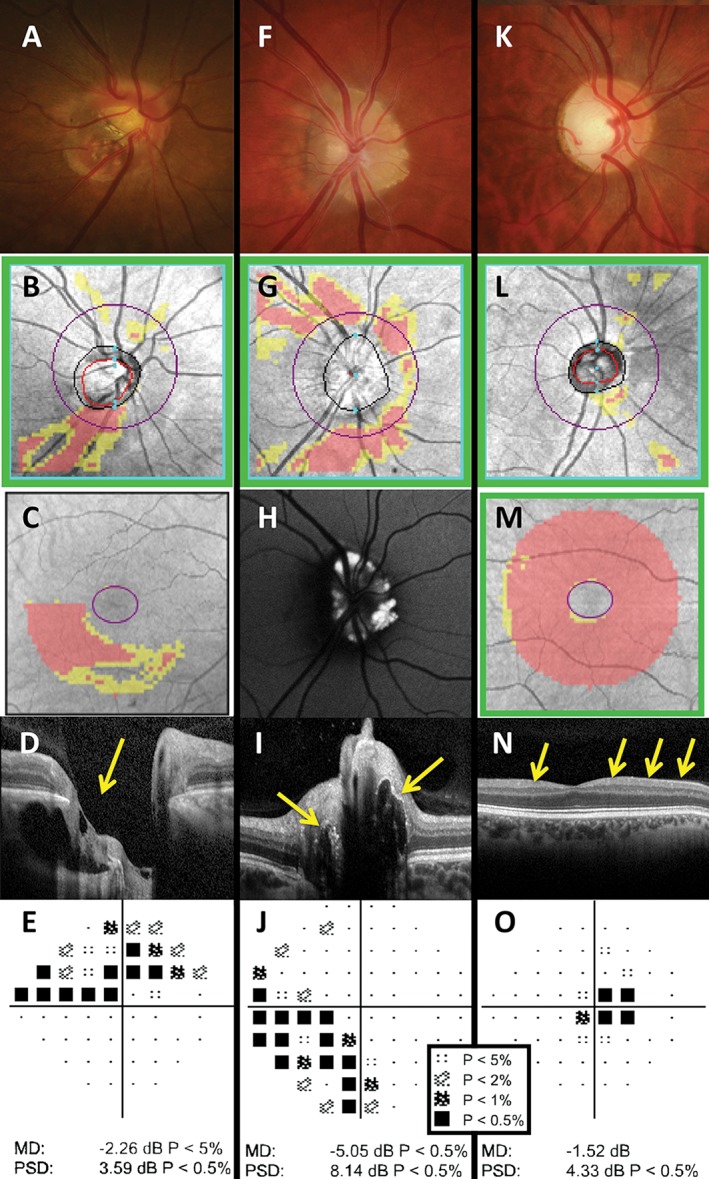
Examples of patients with optic nerve disease: optic disc pit (A–E), optic nerve head drusen (F–J) and dominant optic atrophy (K–O). (A–E) The right eye results of a 50‐year‐old Asian woman who was referred for glaucoma assessment. The optic nerve appeared small, obliquely inserted and tilted, with significant peripapillary atrophy, which has confounded Cirrus optical coherence tomography (OCT) retinal nerve fibre layer (RNFL) analysis (B). The Cirrus Ganglion Cell Analysis (GCA) deviation map showed an inferior arc‐like defect (C) and the Humphrey Field Analyzer (HFA) 24–2 SITA‐Standard visual field result showed a matching superior arcuate defect (E). Coronal scan with the Spectralis OCT allows visualisation of the pit (D, yellow arrow); however, careful inspection of the optic nerve, aided with stereoscopic viewing, showed an optic disc pit in the inferotemporal region, which has caused the visual field defect. The altitudinal‐like visual field loss was unlikely to be due to glaucoma. (F–J) The right eye results of a 31‐year‐old Caucasian woman who was referred for assessment on the basis of raised optic nerve head. The fundoscopic examination showed a heaped optic nerve, although without obscuration of the blood vessels (F). Cirrus RNFL analysis showed thinning of the adjacent RNFL bundles superiorly, inferiorly and nasally (G). Autofluorescence imaging (Spectralis OCT) showed hyperautofluorescence of the optic nerve, especially in the nasal aspect, characteristic of buried optic nerve head drusen (H), with corresponding hyper‐reflective material on the coronal scan (I, yellow arrows).^145^ Although the RNFL appeared reduced superiorly, nasally and inferiorly, the HFA 30–2 SITA‐Standard result showed only an inferonasal depression (J). (K–O) The right eye results of a 39‐year‐old Caucasian man with diagnosed dominant optic atrophy. Fundoscopic examination showed an average‐sized disc with enlarged vertical cup and pallor, particularly of the temporal aspect (K). Cirrus OCT RNFL deviation map showed no significant defects (L) but the GCA deviation map showed a generalised reduction in ganglion cell‐inner plexiform layer thickness across the entire scan area (M). Line scan through the fovea showed marked thinning of the RNFL layer (N, yellow arrows). HFA 24–2 SITA‐Standard was performed, as the visual field defects had a centrocaecal pattern (O). A key for the greyscale levels of probability of normality is shown as an inset.

In comparison to glaucoma and other ischaemic optic neuropathies, other optic diseases, such as inflammatory or compressive neuropathies, can affect different regions of the optic nerve and retinal nerve fibre layer and to varying degrees. Because of this, optic neuropathies such as optic neuritis, neuroretinitis and optic nerve head drusen may all present with different visual field defects.^59^, ^143^, ^144^, ^145^ Therefore, unlike glaucoma, these conditions sometimes do not have obvious structure‐function concordance, unless the region of optic nerve affected by disease is discrete, such as in optic disc pit (Figure 7). Using a 30–2 or similar visual field test grid is often recommended for examining these conditions due to the variability of extent of possible visual field defects.^135^

### Beyond the retina and optic nerve

The anatomy of the optic chiasm and of the visual pathway beyond, means that, in general, visual field defects are bilateral.^146^ Patterns of visual field loss – pre‐chiasmal, chiasmal or post‐chiasmal – are useful for guiding subsequent neuroimaging.^147^, ^148^, ^149^

**Figure 8:**
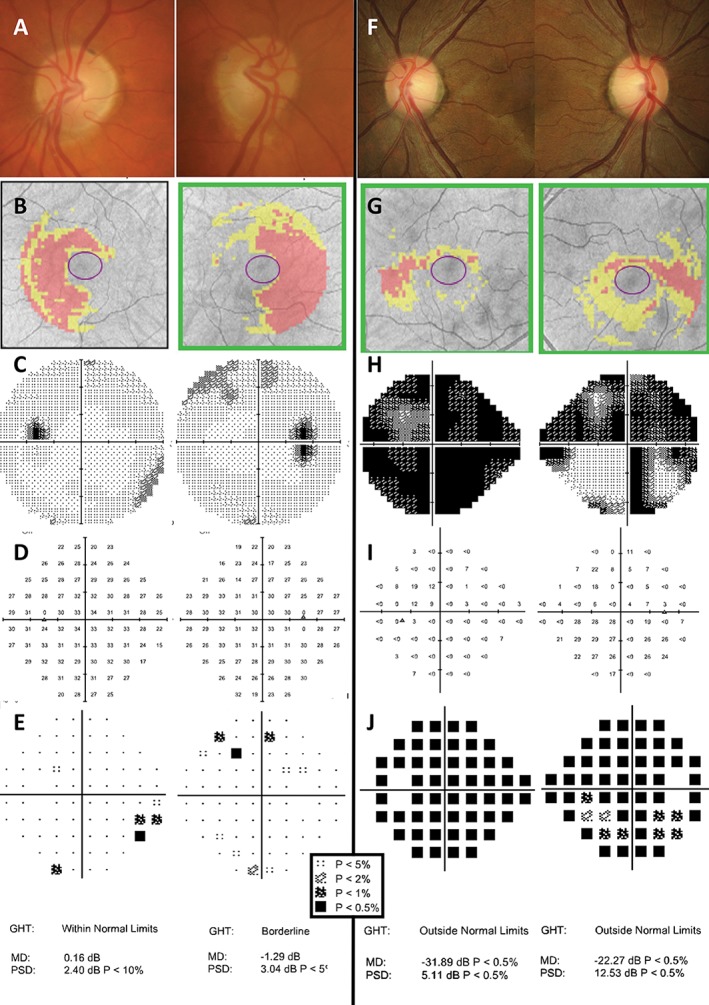
Examples of patients with chiasmal‐type lesions and their visual field defects. Right and left eye fundus photographs and optical coherence tomography (OCT) results have been inverted to portray the visual field results in the conventional method with field on the ipsilateral side, which helps to recognise congruous and symmetrical defects. A key for the greyscale levels of probability of normality for the deviation maps is shown as an inset. (A–E) The clinical findings of a 69‐year‐old Caucasian man with previous pituitary tumour, which had been surgically removed. Fundoscopic examination showed pallor of the temporal neuroretinal rim, right more so than left (A). Cirrus OCT Ganglion Cell Analysis (GCA) deviation map showed thinning in the nasal region in both eyes (B). The Humphrey Field Analyzer (HFA) 30–2 SITA‐Standard results showed isolated clusters of defects that did not follow a typical bitemporal pattern of loss, that is, visual recovery following relief of compression due to the chiasmal lesion (C–E). (F–J) The clinical results of a 27‐year‐old Asian woman who had experienced gradual worsening left vision over the past 2–3 months. Her visual acuities were 6/6^−1^ R and 6/75^+2^ L (no improvement with pinhole). Amsler grid testing showed marked loss of the temporal field, particularly of the left eye. Fundoscopic examination showed temporal pallor of the neuroretinal rim, left more so than right (F). Interestingly, Cirrus GCA deviation map showed only mild depression of the temporal region in both eyes which did not appear that severe (G). HFA 24–2 SITA‐Fast (performed due to patient discomfort on the test) visual field results showed almost complete loss of sensitivity in the left eye and a superonasal and temporal defect in the right eye on the greyscale map (H). In this case, the pattern deviation plots were not useful, due to the extensive amount of visual field loss; these did not reveal a specific neurological pattern of loss (J). Instead, examination of the raw threshold values was more informative (I). In the right eye, there was a distinct change in sensitivity about the vertical midline, particularly inferiorly, with the temporal field exhibiting loss of sensitivity at the level of less than zero dB, in comparison to the near‐normal thresholds of around 27 dB in the nasal region. These findings were typical of a progressive chiasmal lesion, with a particular left‐sided bias (pituitary adenoma confirmed causing anterior chiasmal syndrome).

Chiasmal defects are characteristically bitemporal (Figure 8), with a bias toward either superior (for example, pituitary adenoma) or inferior (for example, craniopharyngioma) bitemporal defects, depending on aetiology.^146^, ^150^ Visual field losses in the far superotemporal and inferotemporal regions of the field may at first be subtle and the wider and symmetrical 30–2 test grid may be required.

**Figure 9:**
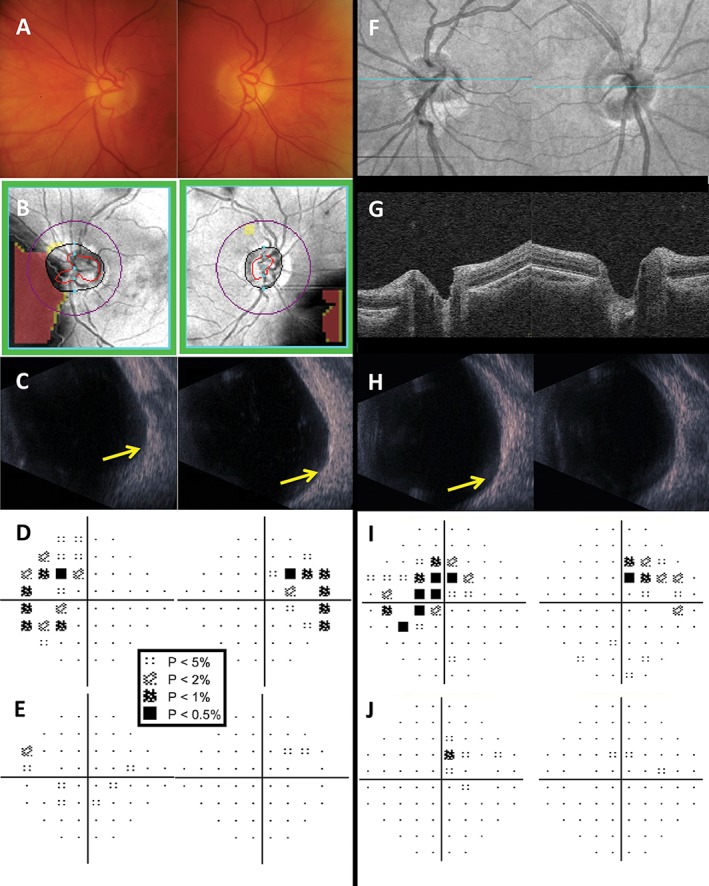
Two examples of patients with tilted disc syndrome. As per the convention of examining visual field results, left eye results are placed on the left and right eye results on the right; hence, the corresponding fundus photographs and optical coherence tomography (OCT) results are also placed on opposite sides to a typical instrument printout. (A) A 76‐year‐old man who was seen for assessment with a suspicious optic nerve head, which appeared tilted and obliquely inserted with clear situs inversus of the blood vessels typical of tilted disc syndrome. Cirrus OCT deviation map results (B) show obvious errors in segmentation of the nasal fundus, as expected of a coloboma in that region. B‐scan ultrasound along the horizontal axis in both eyes show an uneven curvature indicative of a posterior staphyloma in the region of the coloboma (C, yellow arrows). Conventional standard automated perimetry (SAP) testing (Humphrey Field Analyzer [HFA] 24–2 SITA‐Standard) showed a cluster of defects in the nasal region of both eyes which apparently respected the vertical midline (D). The addition of a further −3.25 D lens on top of the patient's near refraction almost completely eliminated the defect, by refocusing rays of light onto the more posteriorly displaced retina (E). Although there was some depression of the nasal, out‐of‐focus portion of the visual field, this did not reach statistical significance. (B) A 27‐year‐old woman with no neurological complaints but progression of myopia in the left eye. Fundoscopic examination showed a more obliquely inserted and tilted disc in the left eye (F, G; note that Cirrus OCT infrared images have been included in lieu of fundus photographs but show the same clinical picture) and subtle coloboma on B‐scan ultrasound (H). In comparison, the right eye showed only a slightly tilted disc (right hand side images). Conventional SAP testing (HFA 30–2 SITA‐Standard) revealed a cluster of defects predominantly in the superotemporal region, left more so than right (I). The addition of a further −3.25 D lens on top of the patient's refraction essentially eliminated the temporal visual field defect (J). Similar to the case shown in (A–E), there was some depression of the nasal, out‐of‐focus portion of the visual field; this did not reach statistical significance. A key for the greyscale levels of probability of normality for the deviation maps is shown as an inset.

One notable differential diagnosis for bitemporal defects is tilted disc syndrome, which may introduce relative myopic defocus (Figure 9).^151^ An additional myopic lens could be used for a repeat visual field test,^151^ which can reduce or eliminate this scotoma (Figure 9). Careful examination of the visual field sensitivity values can give clues as to the diagnosis, which may reduce the need and expense of further testing.

At the optic tract, visual field defects change from being symmetrical about the vertical axis to being homonymous and contralateral to the site of pathology, due to segregation of ipsilateral temporal (uncrossed) and contralateral nasal (crossed) retinal nerve fibre layer bundles. Optic tract and pre‐lateral geniculate nucleus defects tend to be relatively incongruous, due to incomplete pairing of the retinal nerve fibres from anatomically corresponding points in the visual field. In particular, unilateral defects along the optic tract manifest with a relative afferent pupillary defect on the contralateral side, due to asymmetric decussation of the pupillary fibres (approximately 54 to 67 per cent).^152^, ^153^ Defects higher along the visual pathway (for example, cortical lesions) are typically more congruous.^154^, ^155^

**Figure 10:**
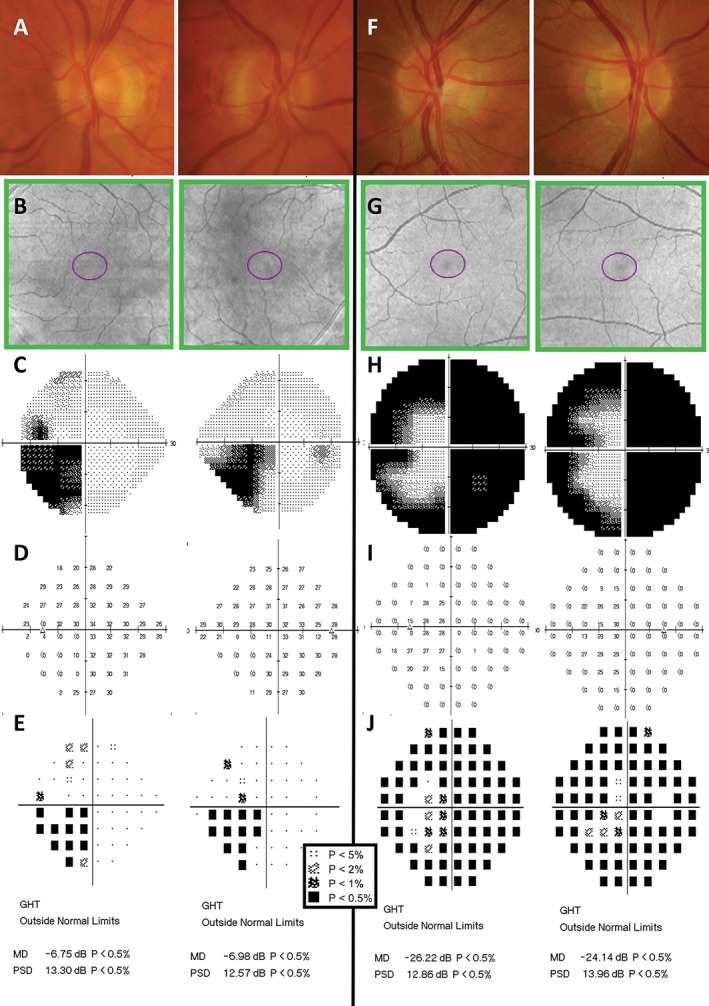
Examples of patients with neurological‐based visual field defects but without optic disc changes (for example, pallor; A, F) or ganglion cell loss on the Ganglion Cell Analysis (GCA) maps (B, G). Humphrey Visual Field Analyzer greyscale (C, H), thresholds (D, I), total deviation map (E) and pattern deviation map (J), with corresponding Glaucoma Hemifield Test, mean deviation and pattern standard deviation results are shown. A key for the greyscale levels of probability of normality for the deviation maps is shown as an inset. (A–E) A 70‐year‐old Caucasian man who was found to have a left inferior homonymous quadrantonopia. (F–J) A 48‐year‐old Caucasian male patient who underwent ophthalmic examination following an episode of occipital lobe cerebral vascular accident seven weeks earlier. There was a right homonymous hemianopia plus constriction of the left superior and inferior fields, sparing the central region of the left field in both eyes. A key for the greyscale levels of probability of normality for the deviation maps is shown as an inset.

Lesions of the axons travelling in the optic radiations or cortical areas post‐lateral geniculate nucleus can give rise to trans‐synaptic retrograde degeneration of the retinal ganglion cells (see Zangerl and colleagues^146^ for a full review). Retrograde degeneration manifests as retinal nerve fibre layer or retinal ganglion cell loss mirroring the visual field defects that respect the vertical midline. Although these patterns are typically concordant with visual field findings, retinal nerve fibre layer and retinal ganglion cell changes on advanced imaging techniques, such as OCT or on optic nerve head examination can take time to develop.^156^ Instead, visual field testing may reveal definitive defects in the absence of significant structural loss (Figure 10).

## THE PROBLEM OF STRUCTURE‐FUNCTION DISCORDANCE

Although there are typical visual field defects that occur with patterns of structural losses in disease, that is, structure‐function concordance, results in reality are often confounded by inherent variability of the measurement technique.^17^, ^157^, ^158^ This is especially true for diseases with slow progression or in the early stages of disease.^159^, ^160^, ^161^ As such, studies in ocular disease often list a requirement for demonstrated repeatable visual field loss, that is, not a reduction of sensitivity due to inherent variability, before classifying the presentation as true disease or progression of disease.^58^, ^159^, ^161^, ^162^

**Figure 11:**
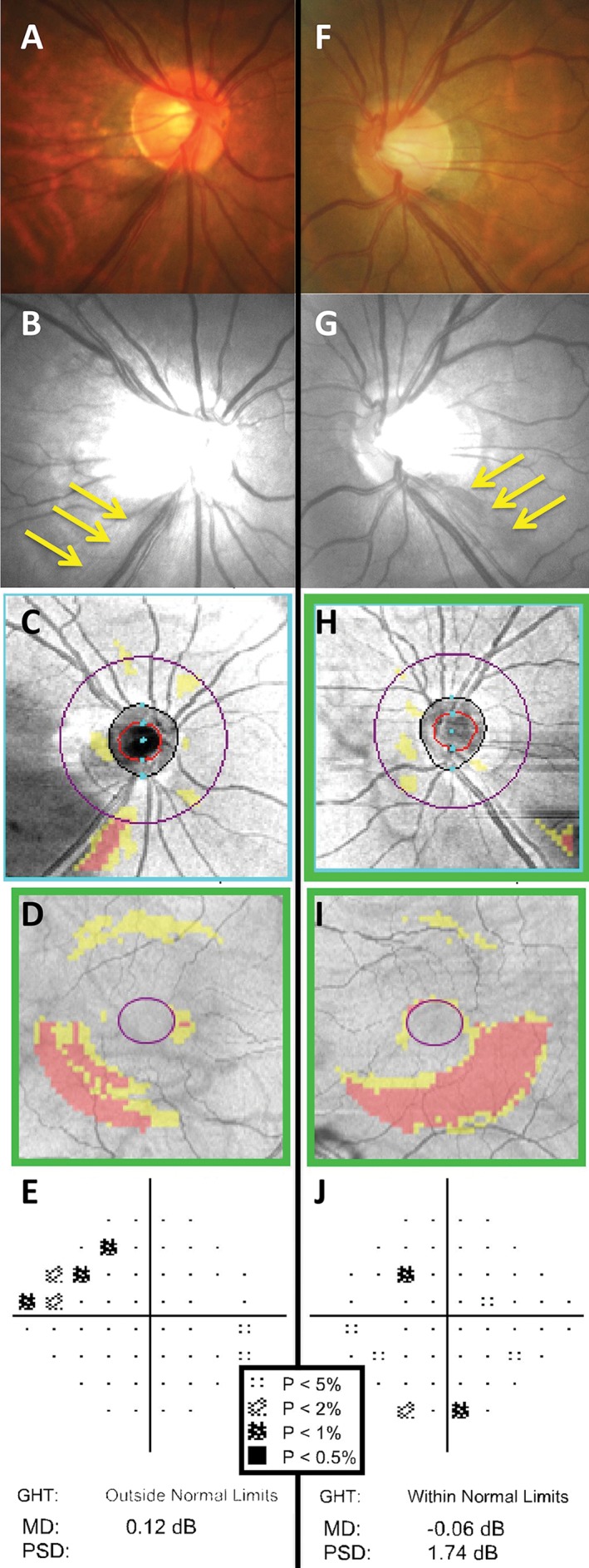
The right (A–E) and left (F–J) eye clinical findings of a 60‐year‐old Asian woman with bilateral glaucoma. A key for the greyscale levels of probability of normality is shown as an inset. Optic nerve head examination showed small‐sized, tilted discs with inferotemporal thinning of the neuroretinal rim (A, F) and corresponding retinal nerve fibre layer (RNFL) loss in both eyes as shown by the yellow arrows (B, G). Cirrus optical coherence tomography (OCT) RNFL deviation map showed more obvious RNFL loss in the right eye (C) compared to the left (H), due to the presence of eye movement artefacts. Cirrus OCT Ganglion Cell Analysis (GCA) deviation map showed inferior arc‐shaped defects of ganglion cell‐inner plexiform layer loss, left (I) more so than right (D). Humphrey Field Analyzer (HFA) 24–2 SITA‐Standard deviation map results showed structural‐function correlation in the right eye, with a superior nasal step (E). The mean deviation (MD) score was 0.12 dB (p > 0.05), the pattern standard deviation (PSD) score was 2.02 dB (p < 0.05) and the Glaucoma Hemifield Test (GHT) was marked as ‘outside normal limits’. In comparison, there was no structure‐function correlation in the left eye, with only isolated points of reduced sensitivity (J). Global indices were also essentially within normal limits: MD score was −0.06 dB (p > 0.05), PSD score was 1.74 dB (p > 0.05) and the GHT was ‘within normal limits’.

While glaucoma has been traditionally defined as an optic neuropathy with corresponding characteristic visual field loss, newer definitions note that statistically significant visual field losses, meeting the criteria set by published research papers, may not necessarily be present, in a stage known as ‘pre‐perimetric glaucoma’ (PPG) (or ‘mild’ glaucoma).^24^ In this case, there is structure‐function discordance, with overt structural deficits and absent visual field loss (Figure 11). PPG presents a diagnostic and management conundrum for clinicians. Waiting for progression prior to the initiation of treatment may mean the development of irreversible visual field loss, while over‐treatment of some patients may reduce overall quality of life.^163^ Although studies have shown that treatment reduces progression rates of patients with pre‐perimetric glaucoma, it is suggested that some patients progress so slowly that early treatment may not be indicated.^22^, ^23^

One of the most frequently quoted statements in ophthalmology^164^ is a variation upon: ‘at least 25 to 35 per cent retinal ganglion cell loss is associated with abnormalities in visual field testing’,^20^, ^21^ with the implication that SAP is relatively insensitive to the loss of neural tissue, accounting for PPG. This is now known to be an incorrect interpretation of the results.^25^, ^165^ Large clinical trials have shown a large variance in the number of patients reaching a visual field endpoint (35 to 86 per cent) for diagnosis or progression of glaucoma prior to the onset of structural change.^58^, ^60^, ^62^ One explanation for this difference is the endpoint definition. For example, the Ocular Hypertension Treatment Study (OHTS) had a visual field endpoint requiring a repeatable defect over three visits spanning six months, with a stringent inclusion criterion of requiring three reliable baseline visual field results.^166^ The European Glaucoma Prevention Study (EGPS) also required three visual field tests but these were spaced closer (within 30 days for confirmation), which may explain in part, why it had a greater proportion of patients reaching a functional endpoint.^167^ The Early Manifest Glaucoma Trial (EMGT) only required two defective visual field results to flag tentative progression, with a criterion that had a lower statistical threshold (three points at p < 0.05, rather than at least one with p < 0.01 in the OHTS).^168^ With less stringent visual field criteria compared to OHTS and EGPS, it follows that EMGT had a greater proportion of patients showing functional loss first.

**Figure 12:**
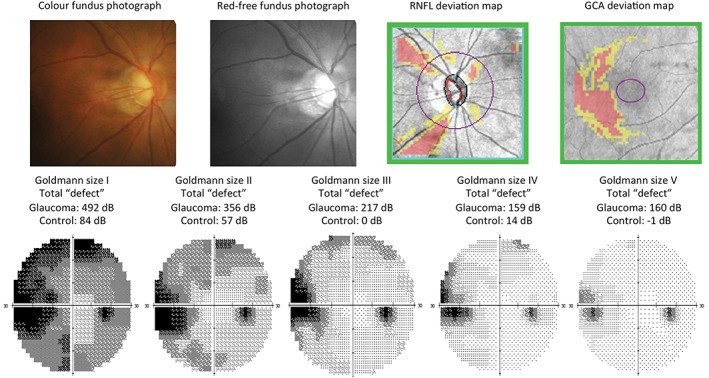
The right eye clinical results of a 58‐year‐old Asian man with normal‐tension glaucoma. Some of this patient's results have been previously reported in Kalloniatis and Khuu^32^ (Table 1, patient E). The fundus examination showed a small disc with clear optic nerve head cupping, superior and inferior neuroretinal rim thinning and retinal nerve fibre layer (RNFL) drop out. Imaging results from the Cirrus optical coherence tomography (OCT) RNFL and Ganglion Cell Analysis (GCA) deviation maps concurred with the fundoscopic examination. Humphrey Field Analyzer (HFA) 30–2 full threshold results for Goldmann sizes I–V are shown below. For clarity in discerning the location and depth of defect, greyscales are shown. Using a standard Goldmann size III stimulus, there was a typical glaucomatous nasal step defect. When using larger stimulus sizes (IV and V), the greyscale appears lighter and smaller in extent, indicative of less visual field loss detected. Conversely, utilising a smaller stimulus size (I and II) shows a wider and deeper extent of visual field loss detected in the nasal region. A central reference point is used in the HFA to depict regions of visual field loss for non‐standard Goldmann sizes (I, II, IV and V) and as such, these are not directly interchangeable with standard size III for comparisons.^8^ Importantly, these total ‘defects’ do not represent a normalised defect, accounting for regional variations across the VF (see: Kalloniatis and Khuu,^32^ Heijl and colleagues^179^ and Russell and colleagues^180^) but rather a coarse comparison with a normal reference and an obvious size‐dependent effect. An age‐matched normal subject's (‘control’) total ‘defect’ results are shown below the results of the glaucoma patient.

**Figure 13:**
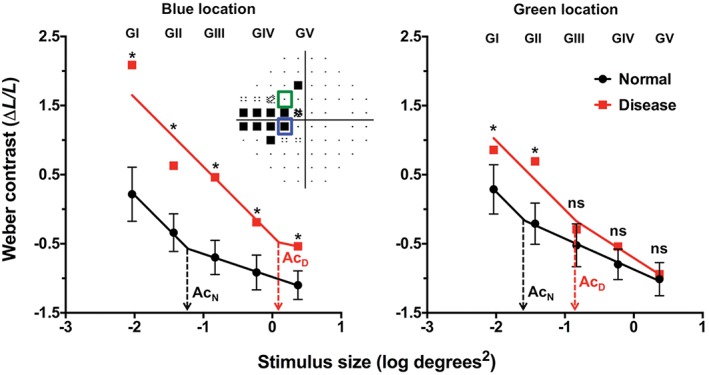
Spatial summation functions for the same patient shown in Figure 12. Humphrey Field Analyzer (HFA) thresholds have been converted into equivalent Weber contrast levels on the y‐axis (as per Khuu and Kalloniatis^5^) with stimulus sizes expressed in log degrees^2^ on the x‐axis. Five points represent the thresholds obtained using each available Goldmann stimulus size on the HFA and the line represents the segmental non‐linear regression with an initial slope fixed at −1, representing the region of complete spatial summation. The dashed lines indicate the critical area (Ac) for normal (black, Ac_N_) and disease (red, Ac_D_) at the two representative locations. The red squares denote the thresholds of the patient with glaucoma and the black circles indicate a group of age‐equivalent normal patients (error bars denote the 95 per cent distribution limits). Two representative locations are shown, coloured coded according to the inset visual field pattern deviation map. For the blue test location, all sizes show a statistically significant elevation in threshold (marked with asterisks) but stimuli that are within complete spatial summation (that is, the slope of −1) have the greatest threshold elevation. For the green test location, only Goldmann sizes I and II had significant threshold elevation (*), while Goldmann sizes III–V were not significant (ns).

Several reasons for discordance in the structure‐function relationship have been suggested, including proposals that SAP is relatively insensitive to early changes in the visual field, a ganglion cell reserve and redundancy^20^, ^169^, ^170^, ^171^ (see Malik, Swanson and Garway‐Heath^25^ for a review of structure‐function models). For example, the use of unequal units (linear versus logarithmic) has been a focal point for research in recent times.^165^ In this vein, there have been suggestions that due to the nature of the units of measurement, SAP is relatively insensitive to early functional loss because of its logarithmic change: it undergoes a relatively ‘flat’ or slow rate of change before manifesting more meaningful progression. Conversely, OCT imaging has been suggested to show more loss at the beginning but plateaus in advanced stages of disease due to the floor effect of its measurement technique (see Table 3 and Figure 4 in Medeiros and colleagues^27^). More recent studies have shown that equating for spatiotemporal summation characteristics can allow detection of functional losses in earlier stages of glaucoma.^32^, ^94^, ^107^ Simple modifications of test stimuli at different visual field test locations can specifically maximise defect detection, while maintaining the widest dynamic range of testing.^32^, ^172^ Defects found with such stimuli have been shown to be commensurate with underlying, detectable structural loss, thereby potentially improving the structure‐function relationship (Figures 12 and 13 and see Figure 4A in Kalloniatis and Khuu^32^).^94^, ^107^, ^172^ These studies highlight the shortcomings of using GIII or GV for detection of visual field defects, which have been suggested previously to maximise the dynamic range and reliability of testing:^101^, ^110^, ^173^ these relatively larger stimuli detect fewer and shallower defects compared to stimuli operating within complete spatial summation. More work is required to determine the optimal stimulus parameters that reveal the maximal threshold elevation in disease, while maximising the dynamic range of the instrument; however, it is likely that future test paradigms may modulate a variety of stimulus parameters, that is, not simply just contrast but also size and duration at various locations in the visual field.

Aside from the sensitivity of perimetric test stimuli, a number of questions remain unanswered. It is still not known whether current methods of measuring ocular structure are the most sensitive for correlating with functional loss, as there are suggestions that retinal ganglion cell dysfunction may contribute to patients with ‘pre‐structural glaucoma’ found using conventional examination techniques.^174^, ^175^ Methods of measuring retinal ganglion cell counts in humans *in vivo* have also been equivocal, showing large variation particularly in normal observers. For example, the position of the retinal nerve fibre layer raphe varies across individuals, which, in conjunction with relatively sparse visual field test grids, confound concordance of measurements.^176^ Individualised structure‐function mapping has been shown to be useful in improving structure‐function correlations but there still exists a range of variables, such as atypical disc appearances that are not accounted for by simple transposition of the OCT map.^126^, ^177^ It is possible that a combination of customised testing paradigms is required for optimal structure and function measurements for disease detection and monitoring for individual patients.^126^, ^127^, ^177^, ^178^

## CONCLUSIONS

While modern technology has improved facets of visual field assessment using standard automated perimetry, the basic psychophysical task of detection of incremental light stimuli has remained virtually unchanged since the 1970s. There is a number of recognised limitations of standard automated perimetry but it remains the clinical standard of assessing the visual field. Recent research has challenged existing test paradigms. In combination with new computational techniques such as accompanying structural measurements, there is growing interest in reconciling the poor structure‐function relationship in early stages of disease. Preliminary results are promising in areas of research attempting to better reconcile the structure‐function relationship but more work is needed to produce a widespread paradigm shift in SAP.
